# β-Glucose-1,6-Bisphosphate Stabilizes Pathological Phophomannomutase2 Mutants In Vitro and Represents a Lead Compound to Develop Pharmacological Chaperones for the Most Common Disorder of Glycosylation, PMM2-CDG

**DOI:** 10.3390/ijms20174164

**Published:** 2019-08-26

**Authors:** Maria Monticelli, Ludovica Liguori, Mariateresa Allocca, Giuseppina Andreotti, Maria Vittoria Cubellis

**Affiliations:** 1 Dipartimento di Biologia, Università Federico II, 80126 Napoli, Italy; 2 Dipartimento di Scienze e Tecnologie Ambientali, Biologiche e Farmaceutiche, Università degli Studi della Campania “Luigi Vanvitelli”, 81100 Caserta, Italy; 3 Istituto di Chimica Biomolecolare—CNR, 80078 Pozzuoli, Italy

**Keywords:** PMM2-CDG, pharmacological chaperone, glucose-1,6-bisphosphate

## Abstract

A large number of mutations causing PMM2-CDG, which is the most frequent disorder of glycosylation, destabilize phosphomannomutase2. We looked for a pharmacological chaperone to cure PMM2-CDG, starting from the structure of a natural ligand of phosphomannomutase2, α-glucose-1,6-bisphosphate. The compound, β-glucose-1,6-bisphosphate, was synthesized and characterized via ^31^P-NMR. β-glucose-1,6-bisphosphate binds its target enzyme in silico. The binding induces a large conformational change that was predicted by the program PELE and validated in vitro by limited proteolysis. The ability of the compound to stabilize wild type phosphomannomutase2, as well as frequently encountered pathogenic mutants, was measured using thermal shift assay. β-glucose-1,6-bisphosphate is relatively resistant to the enzyme that specifically hydrolyses natural esose-bisphosphates.

## 1. Introduction

In humans there exist two phosphomannomutases, PMM1 and PMM2, that interconvert mannose-6-phosphate (M6P) into α-mannose-1-phosphate (αM1P) and to a minor extent, glucose-6-phosphate into α-glucose-1-phosphate (αG1P) [1]. Both enzymes require a sugar bisphosphate, α-mannose-1,6-bisphosphate or α-glucose-1,6-bisphospate, for their mutase activity. Besides being a mutase, PMM1 is phosphatase and plays a prevalent role in the degradation of esose bisphosphate in particular in the presence of inosine monophosphate (IMP) [2,3].

The following scheme summarizes the activities of PMM1 and PMM2.

α-hexose-1,6-P_2_ + PMM ⇆ hexose-6P (or α-hexose-1P) + PMM-P (1)

α-hexose-1P (or hexose-6P) + PMM-P ⇆ α-hexose-1,6-P_2_ + PMM (2)

PMM-P + H_2_O ⇆ PMM + P_i_ (3)

hexose = mannose, (1) + (2) = phosphomannomutase activity
hexose = glucose, (1) + (2) = phosphoglucomutase activity
hexose = mannose or glucose, (1) + (3) = phosphatase activity

Only PMM2 has been associated with human diseases and is responsible for the most common congenital disorder of glycosylation (CDG) [4]. PMM2-CDG, also known as CDG-1A or Jaeken syndrome, is a rare autosomic recessive disease without a cure. The disease is particularly frequent in Scandinavian countries but is pan-ethnically distributed. More than 110 pathological mutations have been associated with the disease [5,6]. A complete absence of PMM2 activity is not compatible with life [7]. Usually, patients are composite heterozygous with one inactivating mutation and one hypomorphic mutation, more rarely they carry two different hypomorphic mutations in heterozygosis or one in homozygosity [8,9]. R141H, which is the most common allele, is inactive because the mutation affects the active site [10], but has never been observed in homozygosity [6].

Pharmacological chaperones (PCs) are low molecular weight molecules that are able to rescue the activity of hypomorphic mutants if the active site is integer [11]. These mutants are intrinsically functional and the low residual activity in the cell is due to their instability. Usually [12], but not necessarily, pharmacological chaperones are non-covalent competitive inhibitors of their target enzymes [13,14,15]. They bind and stabilize mutant enzymes, but natural substrates can compete with them for the active site. In fact, the ultimate test for PC is proving that after the administration to cells, the activity of their target enzyme increases. The genotypes associated with PMM2-CDG retain residual activity and are in principle amenable of treatment with PCs since they all retain residual activity. Even a slight increase in PMM2 activity produced by PCs could alleviate the symptoms of the patients since heterozygous carriers with just 50% normal phosphomannomutase activity in fibroblasts are asymptomatic, patients with approximately 25% normal enzymatic activity exhibit a moderate clinical picture whereas patients with less than 10% normal enzymatic activity present a severe phenotype [16,17]. Common hypomorphic PMM2 mutants are thermo-sensitive, but can be stabilized by low molecular weight molecules [5,10,18,19,20,21]. In general, this has been observed on single mutants, but in some cases, it has been proved in heterozygosity [22].

Some attempts have been carried out to find PC for PMM2-CDG either starting from large collections of commercially available products [19,23] or from known ligands of PMM2 [10,18,22], yet no drug is available.

Perez and coworkers [19] screened 10,000 small molecules from the Myria screen diversity collection and found four hits that did not resemble chemically the natural ligands of PMM2. These molecules were able to stabilize PMM2 mutants in vitro and rescued the activity in human cells. One molecule, 1-(3-chlorophenyl)-3,3-bis(pyridin-2-yl) urea, appeared very appealing for drug development since it did not inhibit PMM2 and did not possess predictable adverse features. Perlestein and co-workers [23] undertook drug repositioning testing 2560 compounds consisting of FDA approved drugs, bioactive tool compounds, and natural products, on a yeast strain whose phophomannomutase gene is defective. Drug repositioning is a very useful approach to look for medicines for rare diseases because, in case of success, it decreases the gap from bench to bedside [24]. Three compounds, α-cyano-4-hydroxycinnamic acid, suramin hexasodium, and 2′,2′-bisepigallocatechin digallate, suppressed growth deficiencies of the defective yeast strain. Their mechanism of action is not known because they were not tested on purified mutant proteins. None of these molecules resembles natural ligands of PMM2. α -cyano-4-hydroxycinnamic acid enhanced PMM2 enzymatic activity in fibroblasts derived from patients and in a nematode model of PMM2-CDG [25]. Since α -cyano-4-hydroxycinnamic acid shares the carboxylic acid-containing pharmacophore of aldose reductase inhibitors, Perlestein and co-workers [25] tested other commercially available inhibitors of the same enzyme in the nematode model and fibroblasts. They found that epalrestat, which is a safe, orally bioavailable, and brain penetrant aldose reductase inhibitor used to treat diabetic peripheral neuropathy, rescued PMM2 enzymatic activity in both species. Epalrest is a monocarboxylic acid and contains a phenyl and a rhodanine group and does not resemble chemically PMM2 ligand. Direct binding and stabilization to mutant PMM2 proteins is not among the modes of actions proposed by Perlestein and co-workers for epalrest [25].

α-D-Glucose 1,6-bisphosphate (αG16P) could be considered an effective chaperone to rescue unstable pathological variants because it binds PMM2, induces the closure of the enzyme, raises the melting temperature, and activates catalysis [10,18,22]. However, it is rapidly hydrolyzed by PMM1 in particular in the presence of IMP [2,3].

In this paper, we describe a novel approach. We synthesized a molecule, β-glucose-1,6-bisphosphate (βG16P) that is an analog of a natural ligand of PMM2, αG16P. βG16P and αG16P were fully characterized by ^31^P-NMR. This technique can be very useful to measure the activity of PMM1 and PMM2 directly without the aid of ancillary enzymes. In silico observations using the program PELE [26] show that βG16P induces a large conformational change in the structure of PMM2 and the closure of the active site. βG16P can bind PMM2 and stabilize the wild type and hypomorphic PMM2. As it is the case for the majority of PCs used so far for other diseases, βG16P is an inhibitor of its target enzyme [12]. Compared to αG16P, the β anomer is more resistant to the hydrolysis by PMM1.

## 2. Results

### 2.1. Synthesis and ^31^P-NMR Characterization of βG16P

**Figure 1:**
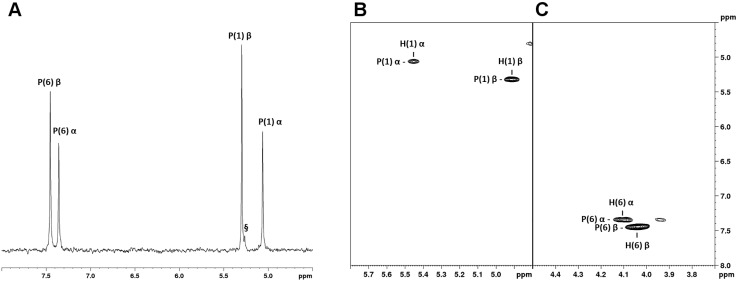
^31^P-NMR characterization of α-D-Glucose 1,6-bisphosphate (αG16P) and β-glucose-1,6-bisphosphate (βG16P). (**A**) ^1^H-decoupled one-dimensional ^31^P spectrum. (**B**,**C**) Selected regions of the ^1^H-^31^P HSQC spectrum. Both the spectra were acquired in H_2_O + D_2_O 10% in the presence of EDTA 50 mM; ppm were referred to creatine phosphate (0 ppm, ^31^P scale) and to trimethylsilylpropanoic acid (0 ppm, ^1^H scale). P(6) and P(1) indicate the positions of phosphorous nuclei in the molecules; §: inorganic phosphate.

βG1P was synthesized from maltose with bacterial phosphorylase and phosphorylated with phosphofructokinase to generate the bisphosphate sugar (about 40 mg were obtained starting from 650 mg of maltose). βG16P was analyzed by ^31^P-NMR spectroscopy and compared to commercial αG16P. ^1^H-decoupled one-dimensional ^31^P spectrum shows two signals, one at 5.30 pmm and one at 7.45 ppm, for the P nucleus in the position 1, P(1), and in position 6, P(6) respectively (Figure 1A). Both signals are deshielded compared to those of the α anomer (5.06 and 7.36 ppm). 2D HSQC ^1^H-^31^P spectra were recorded for both anomers. The signals of βG16P are clearly resolved and distinguishable from those of αG16P (Figure 1B,C).

### 2.2. βG16P Binds PMM2 and Induces a Conformational Change

**Figure 2:**
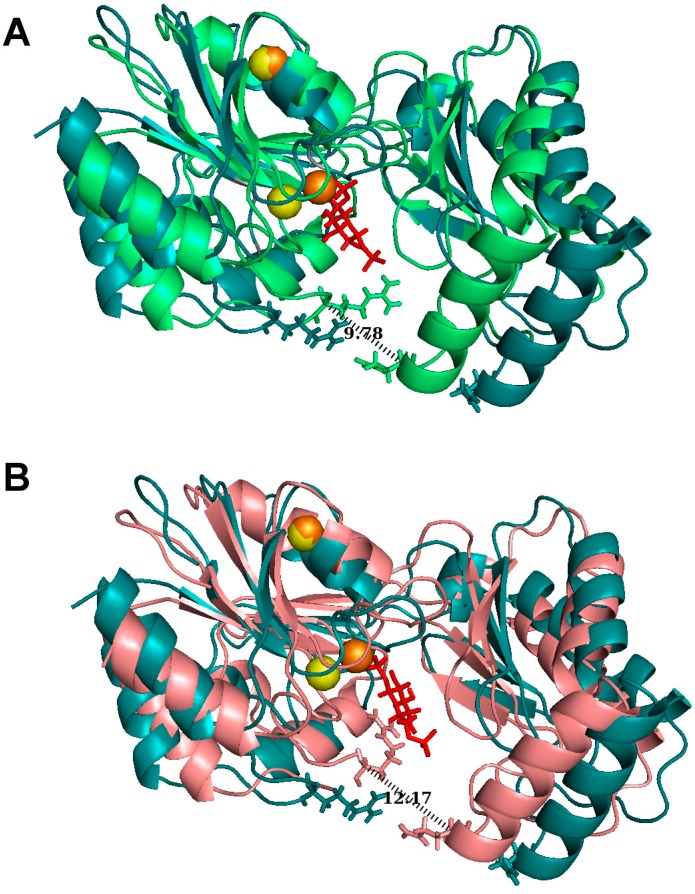
In silico binding models for PMM2 and βG16P: either the phosphate proximal to C6, P6_Mg mode (**A**), or the phosphate proximal to C1, P1_Mg mode (**B**), can interact with the catalytic site. PMM2 in the initial state is in cyan (**A**,**B**); the two closed conformations are in green (**A**) and pink (**B**); βG16P is red; Mg^2+^ are shown as spheres.

**Figure 3:**
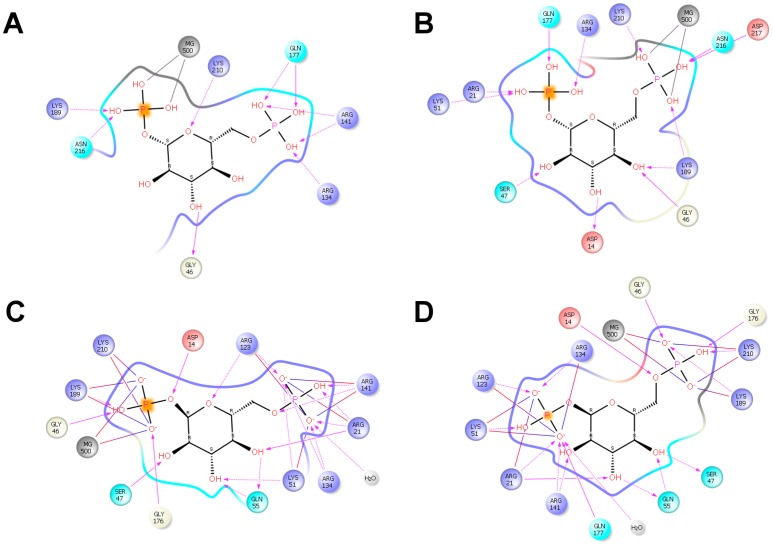
Interactions between PMM2 and ligands: residues that interact with βG16P by hydrogen bonds or salt bridges in P1_Mg mode (**A**) and P6_Mg mode (**B**) respectively; residues that interact with αG16P by hydrogen bonds or salt bridges in P1_Mg (**C**) mode and P6_Mg mode (**D**) respectively.

In silico docking was carried out to test the binding of βG16P onto PMM2 with the program PELE [26]. PMM2 is a homodimer and each subunit is made up by a core (res 1–81; 189–247) and a cap (res 86–185) domain, connected by hinge peptides. The structure of the enzyme is deposited in the PDB with the code 2AMY. Due to the absence of a few atoms and to some disordered regions in 2AMY, the structure cannot be used as such for in silico docking. In a previous paper of ours, we described how 2AMY can be fixed and a native-like model can be generated [10]. The native-like model, which is in open conformation as 2AMY, was used to carry out in silico docking of βG16P. The ligand was initially placed far from the active site (~30 Å) and was free to explore the protein surface with no bias. After a global search, a refinement was carried out. The binding of βG16P binding into the active site induces a large backbone motion and the closing up of PMM2. Actually, we observed two binding modes, in one case the phosphate at position 1, P(1), interacts with the catalytic site (Asp12, Asp14, and Asp 217 that form an acidic triad coordinated with Mg^2+^), in the other case, it is the phosphate at position 6, P(6) to interact with the catalytic site. We will refer to the first case as P1_Mg mode and the second case as P6_Mg mode. In Figure 2 we show the initial unbound PMM2 structure and the two bound structures. It can be observed that the closure is tighter in the P6_Mg mode than in P1_Mg mode. In Figure 3A,B, we show the residues that interact with βG16P by hydrogen bonds or salt bridges in P1_Mg mode and P6_Mg mode respectively.

For comparison, we show the interactions that were observed when αG16P was docked onto the same PMM2 native-like structure (Figure 3C,D) with the same protocol [10]. PMM2 forms fewer contacts with βG16P than with αG16P, in particular in P1_Mg mode, and Asp14 of acidic triad does not come in contact with the phosphate. Upon βG16P binding, the core and the cap domain close up and the distance between Arg21 and Gln138 alpha carbons experiences the largest change. The number of contacts correlates with the tightness of the closure of PMM2. In fact, we measured that the distance between Arg21 and Gln138 alpha carbons is 12.2 and 9.8 Å for βG16P respectively in P1_Mg mode and P6_Mg mode and 7.7 and 7.4 for αG16P in P1_Mg mode and P6_Mg mode respectively.

**Figure 4:**
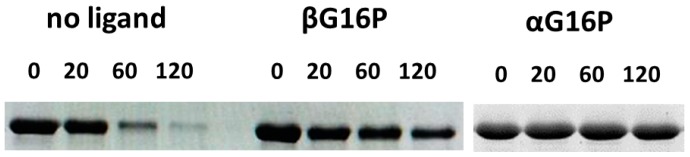
Limited proteolysis of wild type PMM2 by trypsin. Wild type PMM2 was incubated at 37 °C with trypsin in a 50:1 ratio in the presence or the absence of βG16P or αG16P 0.5 mM. Aliquots were withdrawn at specified times (0, 20, 60, 120 min) and analyzed by SDS-PAGE and Coomassie staining.

When the core and cap domains close up, the solvent accessibility of some residues changes. In particular, the exposure of Arg21 to solvent decreases dramatically passing from 98% in the open model to 15% in the closed model. The conformational change that is observed in silico can be tested by limited proteolysis with trypsin. Under the conditions used in the experiment, neutral pH NaCl 150 mM, it had been demonstrated that wt-PMM2 is a dimer [18]. In Figure 4 we show the results obtained incubating wt-PMM2 with the protease in the absence or in the presence of ligands and demonstrate that βG16P binding protects PMM2 from the protease as well as αG16P possibly by rendering the enzyme more compact. The results obtained by SDS-PAGE are qualitative but were observed in three independent experiments. The conformational change induced by αG16P has already been discussed in a previous paper of ours [10].

### 2.3. βG16P Inhibits PMM2

PMM2 has two enzymatic activities since it acts as a phosphomannomutase (interconversion of αM1P and M6P) or as phosphoglucomutase (interconversion of αG1P and G6P) and in both cases a bis-phosphate sugar activator is needed [1,18].

**Figure 5:**
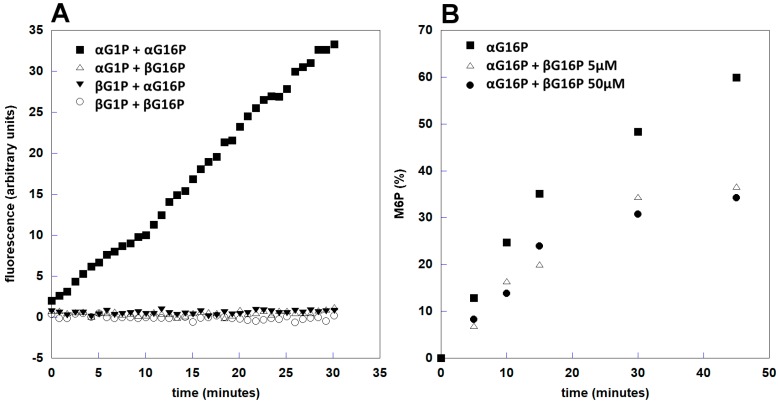
PMM2 phosphoglucomutase activity monitored by fluorescence spectroscopy (**A**) and phosphomannomutase activity monitored by ^31^P-NMR (**B**). (A) A total of 0.08 μg PMM2 were incubated at room temperature with different combinations of α or βG1P as substrates and α or βG16P as activators, in the presence of G6PDH and NADP+. (B) A total of 0.12 μg PMM2 were incubated at 32 °C with 1 mM α-mannose-1-phosphate (αM1P); αG16P 5 μM was used as an activator, in the absence or the presence of βG16P 5 or 50 μM.

Standard enzymatic tests for PMM2 require the use of ancillary enzymes. Dosing phosphoglucomutase activity is simpler and requires only glucose6P dehydrogenase to monitor the formation of G6P measuring the production of NADPH spectrophotometrically or fluorimetrically. We carried out the fluorimetric assay of phophoglucomutase activity using different pairs of substrates and activators. In Figure 5A, it can be observed that βG1P is not a substrate (βG1P + αG16P) and that βG16P is not an activator (αG1P + βG16P). The controls were carried out employing only beta (βG1P + βG16P) or only alpha anomers (αG1P + αG16P).

Standard spectrophotometric tests of mannomutase activity are more complex and require three ancillary enzymes to generate NADPH. Alternatively, the formation of M6P and the consumption of αM1P can be monitored directly by recording ^31^P-NMR spectra.

These experiments require the accumulation of several scans for each NMR spectrum to have a good S/N ratio. They cannot be used to measure initial velocities, but show clearly all the phosphorylated species present in the solution and permit to avoid the usage of ancillary enzymes. These experiments confirm that βG16P is not an activator and show that it inhibits PMM2 (Figure 5B).

### 2.4. βG16P Stabilizes PMM2

**Figure 6:**
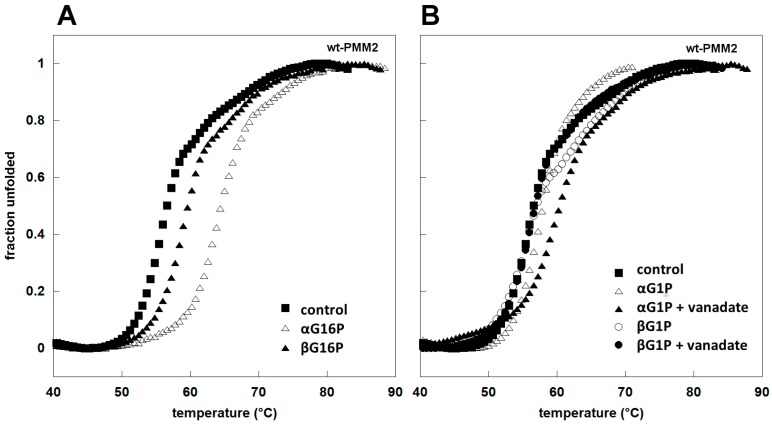
Thermal stability in the presence of ligands. wt-PMM2 0.3 mg/mL was incubated with different ligands 0.5 mM ((**A**) αG16P and βG16P; (**B**) αG1P and βG1P, with and without vanadate); the melting curves were measured in the presence of dithiothreitol (DTT) 1 mM and Sypro Orange 2.4x, from 20 to 90 °C with increments of 1 °C/min.

We carried out a thermal shift assay to test the ability of βG16P to work as a chaperone, i.e., to stabilize PMM2. The effects of αG16P and βG16P were compared (Figure 6A). It can be observed that both anomers stabilize PMM2 although the extent of the stabilization is higher for αG16P: the increase of the melting temperature was about 4 °C in the presence of βG16P (from 54.8 ± 0.3 to 58.5 ± 0.5 °C) and about 9 °C in the presence of αG16P (from 54.8 ± 0.3 to 63.4 ± 0.4 °C).

αG1P is a stabilizer of PMM2 too. Its effect is strengthened by vandate, an inhibitor of PMM2 which mimics phosphate and recreates a non-covalent complex with αG1P similar to sugar 1,6-bisphosphate in the active site. βG1P does not stabilize PMM2 either in the absence or in the presence of vanadate (Figure 6B). This observation and the lack of activity (Figure 4) suggests that βG1P does not bind PMM2.

**Figure 7:**
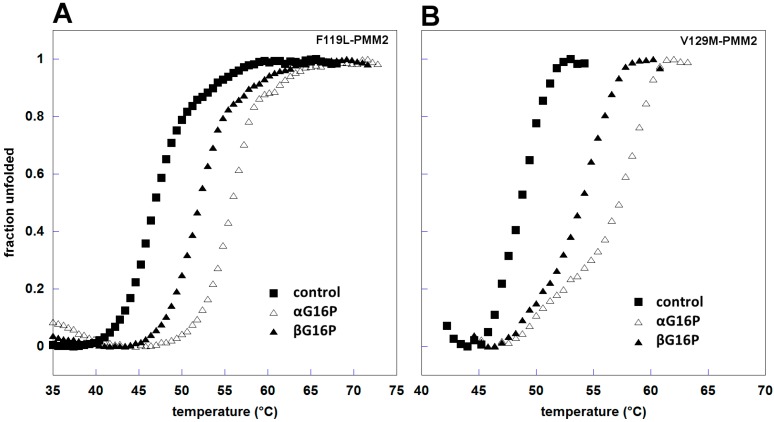
Thermal stability of pathological mutants in the presence of ligands. F119L-PMM2 (**A**) and V129M-PMM2 (**B**) 0.3 mg/mL were incubated with different ligands, αG16P (0.5 mM), and βG16P (0.5 mM). The melting curves were measured in the presence of DTT 1 mM and Sypro Orange 2.4x, from 20 to 90 °C with increments of 1 °C/min.

We also tested the effect βG16P on two pathological mutants, F119L and V129M. Overall, βG16P 0.5 mM caused an increase of about 4 °C, from 46.1 ± 0.4 to 50.3 ± 0.4 °C, while αG16P at the same concentration produces an increase of about 8 °C, up to 54.2 ± 0.8 °C. As far as V129M-PMM2 is concerned the melting temperature in the presence of βG16P increases of about 7 °C, from 47.3 ± 0.4 to 53.9 ± 0.4 °C, and of about 10 °C in the presence of αG16P, up to 58.1 ± 1.3 °C (Figure 7B).

Cell viability tests showed that βG16P is well tolerated by cells when administered at 0.25 or 0.5 mM.

### 2.5. βG16P is a Poor Substrate of PMM1

αG16P is degraded rapidly by PMM1 in particular in the presence of IMP [2,3]. We tested the stability of βG16P to the hydrolysis by PMM1 both in the absence and in the presence of IMP using ^31^P-NMR. It is very convenient that the signals of αG16P and βG16P can be clearly distinguished in the spectra. Hence, the experiment can be carried out by incubating both bisphosphates with PMM1 and examining whether or not only the alpha anomer is hydrolyzed.

The results are summarized in Table 1. PMM1 hydrolyses αG16P preferentially and the different susceptibilities of the two anomers are larger in the presence of IMP.

It was also noticed that the hydrolysis of αG16P produces Pi, αG1P, or G6P conversely the hydrolysis of βG16P produces Pi and βG1P only, suggesting that βG1P is not a substrate of PMM1 (data not shown).

## 3. Discussion

Although several therapeutic approaches for PMM2-CDG [27,28] have been proposed including mannose supplementation [29,30], membrane-permeable α-mannose-1-P [31,32], and the increase mannose-6-P flux into glycosylation pathways [33], metformin [34] and acetazolamide [35], a drug addressing unstable PMM2 mutants is not yet available.

In many cases, the reduced activity of PMM2 hypomorphic mutants is a consequence of their reduced intracellular concentration that in turn is the consequence of the clearance of unstable proteins by the quality control systems of the cell. Using the program SDM [36] we could predict that 70% of pathological mutations are destabilizing. In such cases, PCs represent a good choice for patients [19].

There are two approaches to find pharmacological chaperones. The first one requires the screening of large libraries of molecules. The effort, which is big both in terms of time and budget, does not guarantee finding a drug ready for clinical trials but may produce a lead compound that requires further maturation. The other approach requires an educated guess. One can start from the structures of known ligands of the enzyme and try to modify them to improve their potency, their safety, and their half-life. Although the second approach may seem reductive, it has produced drugs for other pathologies that have been approved and introduced into clinical practice [37,38]. In this paper, we applied the second approach. We synthesized an analog of αG16P, which is a natural ligand of PMM2. αG16P itself could be considered an effective chaperone to rescue unstable pathological variants because it binds PMM2, induces the closure of the enzyme, raises the melting temperature, and activates catalysis. Regrettably, αG16P is hydrolyzed by PMM1 in particular in the presence of IMP [2,3]. Although data concerning the half-life of αG16P in vivo are not available, it can be predicted that its hydrolysis by PMM1 can hinder its utility as a drug. For this reason, we explored the possibility of employing an analogous bisphosphate sugar that would not be degraded as easily as αG16P by PMM1.

We synthesized the anomer βG16P and characterized it by NMR. ^31^P-NMR proved to be a useful technique because it consents to monitor most phosphorylated metabolites and to distinguish αG16P from βG16P anomers. We proved that βG16P binds PMM2 in silico. Incidentally, our experiments confirm the usefulness of PELE [26] as a program for in silico docking. In fact, the number of contacts and the extent of closure predicted by PELE for the two anomers are fully in line with the experimental results.

βG16P is a “classical” PC since it is a mild non-covalent inhibitor of PMM2. Usually, the chaperoning effect of drugs is tested in vitro on wild type enzymes. In the case of PMM2 some mutants can be expressed in *E. coli* and purified. Hence the chaperoning effect of βG16P can be tested on pathological mutants as well as on the wild type enzyme. We have chosen two mutants, F119L and V129M. Particularly, F119L is a very frequent variant and it has been observed both in association with R141H and in homozygosis [8,9,39]. Since F119 does not occur in the active site, the mutant is active although less stable than the wild type. V129M is relatively common in Italy where it has been observed in association with R141H [40]. Position 129 is not located in the active site and the mutant retains more than 50% activity but is less stable than wt-PMM2 [41]. F119L and V129M are amenable for the cure with pharmacological chaperones in principle. We could prove that βG16P stabilizes pathogenic mutants. βG16P is a poor substrate of PMM1 and for this reason, it can be preferred to αG16P as a PC. We are confident that the pharmacokinetics of βG16P can be superior to those of αG16P. The next step of the process that starts from the known ligand, would be enhancing its bioavailability reducing the charges by chemical modification with nontoxic acetoxymethyl groups as proposed for mannose-1 phosphate [31] and using the hydrophobic derivative βG16P as a prodrug. Once inside the cell, the modifying groups would be hydrolyzed generating βG16P. Another possibility to facilitate the entrance of βG16P is offered by liposomes as proposed by Glycomine [42]. The stabilizing effect of βG16P (through the usage of hydrophobic derivatives or with liposomes) could be tested on fibroblasts derived from patients either measuring the increase of PMM2 activity or monitoring the normalization of N-glycosylated biomarkers [28].

## 4. Materials and Methods

### 4.1. Materials

α-D-Glucose 1,6-bisphosphate potassium salt hydrate, α-D-Glucose 1-phosphate disodium salt hydrate, α-D(+)Mannose 1-phosphate sodium salt hydrate, Inosine 5′-monophosphate disodium salt hydrate, Adenosine 5′-triphosphate disodium salt hydrate, Maltose phosphorylase from *Enterococcus* sp., Fructose-6-phosphate Kinase from *Bacillus stearothermophilus*, Trimethylamine, 3-(4,5-dimethylthazol-2-yl)-2,5-diphenyltetrazolium bromide were purchased from Sigma-Aldrich (Sigma-Aldrich, Milan, Italy). Trypsin was purchased from ICN Pharmaceuticals (MP Biomedicals Germany GmbH).

AG1x8 Resin 200-400 Mesh Hydroxide Form was purchased from Bio-Rad (Bio-Rad Laboratories Srl, Milan, Italy).

Sypro Orange was from Invitrogen Molecular Probes (Invitrogen Molecular Probes, Monza, Italy), StepOne^TM^ Real Time PCR System from Applied Biosystems (Applied Biosystems, Foster City, CA, USA), strips from Sarstedt (Multiply–μStrip Pro 8-strip low profile) (Sarstedt Srl, Milan, Italy).

D(+)Maltose monohydrate, β-Nicotinamide adenine dinucleotide phosphate sodium salt, Glucose-6-phosphate Dehydrogenase, Creatine phosphate disodium salt thetrahydrate were from Alfa Aesar (Alfa Aesar, Thermo Scientific, Kandel, Germany).

### 4.2. Synthesis of α-Glucose-1-Phosphate and β-Glucose-1,6-Bisphosphate

β-glucose-1-phosphate and β-glucose-1,6-bisphosphate were prepared as described by [43] with minor changes. Briefly, for β-glucose-1-phosphate a solution containing maltose 1 M, potassium phosphate 100 mM pH 7.5, MgCl_2_ 2 mM was incubated with maltose phosphorylase 5 U/mL for 2 h at room temperature. Synthesis of βG1P was verified by ^31^P-NMR and the concentration of βG1P was estimated by comparison with an internal standard. β-glucose-1,6-bisphosphate was produced incubating βG1P and ATP-Mg^2+^ (1:1 ratio) with phosphofructokinase 15 U/mL overnight at room temperature in a buffer containing potassium phosphate 25 mM, Hepes 14 mM pH 7.5.

βG1P and βG16P were purified on an AG1x8 Hydroxide Form column equilibrated with 0.01 M triethylammonium bicarbonate pH 7.5. A step gradient of triethylammonium bicarbonate from 0.01 M to 1 M was used to elute the sugars; fractions (lyophilized and dissolved in water) were analyzed by ^31^P-NMR. βG1P was eluted at 0.3–0.4 M, βG16 was eluted at 0.8–0.95 M. Triethylammonium bicarbonate 1 M was prepared by bubbling CO_2_ gas into a triethylammine 1 M solution until reaching the desired pH.

### 4.3. ^31^P-NMR Spectroscopy

A Bruker AVANCE^TM^III HD spectrometer 400 MHz, equipped with a BBO BB-H&F-D CryoProbeTM Prodigy fitted with a gradient along the Z-axis, was used for the NMR analysis (Bruker Italia Srl, Milan, Italy).

The ^1^H-decoupled one-dimensional ^31^P (zgpg) spectra were recorded at 161.976 MHz; spectral width 120 ppm, delay time 1.2 s, pulse width of 12.0 μs were applied.

The phase-sensitive 2D HSQC using Echo/Antiecho-TPPI gradient selection, with decoupling during acquisition, (hsqcetgp) ^1^H-^31^P spectra were recorded at 400.13/161.97 MHz; spectral width 32 ppm, delay time 1.2 sec, pulse width of 12.0 μs, frequency offset of 2nd nucleus −4.0 ppm were applied. Coupling constant was 120 Hz; function type was non-uniform sampling, with a NUS amount of 70%.

### 4.4. Docking and Structure Analysis

In silico docking of βG16P was carried out as described for αG16P [10].

Briefly two different exploration runs were performed: (a) a global free search and (b) a local refinement.

(a) The global search was performed by combining a long (6 Å) and a short (1.5 Å) ligand perturbation steps, with a 75%/25% probability, respectively. Rotations were kept in the [0°–90°] range. A randomly chosen search direction was kept for two Monte Carlo steps, allowing a more complete exploration of the entire protein surface. No information about the bound structure was used to drive the search. Anisotropic normale mode perturbation included the lowest six modes, with maximum displacements of the alpha carbon of 1 Å. Within the lowest six modes, a randomly chosen mode was kept for six steps to facilitate large conformational exploration.
(b) The local search used translations of only 0.5 A and rotations in the [0°–180°] range. Furthermore, to keep the ligand in the active site, random search direction was maintained to only one iteration.

Residue percent accessibility was calculated with PISA (PISA v1.48 European Bioinformatics Institute, Hinxton, UK) [44]. Active site residues were identified with DrosteP [45]. The figure of superimposed proteins was prepared with PyMOL (PyMOL 2.3.0 Schrödinger, LLC, New York, NY, USA) [46]. Ligand protein interactions were drawn with Maestro (Maestro, Schrödinger Release 2015-2: Maestro, Schrödinger, LLC, New York, NY, USA, 2015) [47].

### 4.5. Protein Expression and Purification

wt-PMM1 [3,48], wt-PMM2 and its mutants (F119L-PMM2 and V129M-PMM2) [18,41] were expressed and purified as already described.

All the PMMs were expressed (using the vector Pet22b+) in *E. coli* BL21(DE3) strain grown at 37 °C in LB broth containing ampicillin 0.2 mg/mL. The expression of wt-PMM1 was performed by adding IPTG 0.4 mM when the optical density was 0.5 and prolonging the incubation for 4 h after induction. Bacteria were then harvested, washed with PBS, suspended in Hepes 50 mM pH 7.5 (containing 2-mercaptoethanol 0.1 mM, EDTA 1 mM, phenylmethylsulfonyl fluoride 0.1 mM), and enzymatically lysed with lysozyme 1 mg/mL, treated with Deoxyribonuclease I 0.005 mg/mL after adding MgCl_2_ 10 mM, and centrifuged. Ammonium sulphate was added to the clear homogenate up to 50% saturation. The precipitate was recovered, dissolved, dialyzed (in Hepes 50 mM pH 7.1, MgCl_2_ 5 mM, 2-mercaptoethanol 1 mM), and fractionated on a DEAE-Sepharose ff with a salt gradient (0–0.7 M NaCl in Hepes 50 mM pH 7.1, MgCl_2_ 5 mM, 2-mercaptoethanol 1 mM). A subsequent fractionation step was conducted on a Butyl-Sepharose ff column (equilibrated in Tris 50 mM pH 7.1 containing MgCl_2_ 5 mM, 2-mercaptoethanol 1 mM, ammonium sulphate 20%). Ammonium sulphate 20% was added to the sample and then loaded onto the column, the proteins were eluted with a gradient 20%–0% ammonium sulphate. The active fractions, judged pure by SDS-PAGE, were dialyzed (in Hepes 20 mM pH 7.5, MgCl_2_ 1 mM, NaCl 150 mM), concentrated, and stored at −20 °C.

The expression of PMM2s (wt and mutants) was performed by adding IPTG 0.4 mM when the optical density was 0.5 (V129M) or 0.8 (F119L and wt) and prolonging the incubation for 4 h after induction. Bacteria were harvested, washed with PBS, suspended in Tris 50 mM, pH 7.5 (containing 2-mercaptoethanol 1 mM, EDTA 5 mM, and phenylmethylsulfonyl fluoride 1 mM), and enzymatically lysed with lysozyme 1 mg/mL, treated with Deoxyribonuclease I 0.005 mg/mL after adding MgCl_2_ 10 mM, then centrifuged. Ammonium sulphate was added to the clear homogenate up to 60% saturation. The precipitate was recovered, dissolved, and dialyzed (in Hepes 50 mM pH 7.1 containing 5 mM MgCl_2_ and 2-mercaptoethanol 1 mM), then loaded on a DEAE-Sepharose ff column. The pass-through was collected, concentrated, and subsequently fractionated on a Superdex 75 column (in Hepes 20 mM, MgCl_2_ 1 mM, NaCl 150 mM, pH 7.5). The active fractions, judged pure by SDS-PAGE, were pooled, concentrated, and stored at −20 °C.

### 4.6. Limited Proteolysis

Limited proteolysis is a useful assay to test PCs [49]. wt-PMM2 to a final concentration 0.3 mg/mL was incubated with trypsin in a 50:1 ratio, in Hepes 20 mM pH 7.5, MgCl_2_ 1 mM, NaCl 150 mM, in the presence or the absence of βG16P or αG16P 0.5 mM. The incubation was carried out at 37 °C for 120 min, collecting samples at specified time intervals. The reaction was stopped by addition of sample buffer up to Tris 50 mM pH 6.8, 10% glycerol, 2% SDS, dithiothreitol (DTT) 100 mM, 0.1 bromophenol blue, then boiling for 5 min and quick cooling. Samples were analyzed by SDS-PAGE.

### 4.7. Enzyme Assay by Fluorescence Spectroscopy

Phosphoglucomutase activity was measured recording the reduction of NADP+ to NADPH. 0.08 μg PMM2 were incubated at 25 °C in a solution containing Hepes 20 mM pH 7.5, MgCl2 5 mM, G6PDH 2.6 U/mL, NADP+ 0.25 mM, BSA 0.1 mg/mL. αG1P or βG1P 40 μM were used as the substrate; αG16P or βG16P 27 μM were used as the activator. Fluorescence at 340/445 nm (ex/em) was recorded for 30 min using a Varian Cary Eclipse Fluorescence Spectrophotometer equipped with a Microplate Reader (Agilent Technologies Italia SpA, Milan, Italy).

### 4.8. Enzyme Assay by ^31^P-NMR

Enzymatic activities of phosphomannomutases (phosphomannomutase and phosphatase activities) can be analyzed recording ^31^P-NMR spectra [3]. Both these activities were measured in Hepes 20 mM pH 7.3, MgCl2 1 mM, creatine phosphate (CP) 0.5 mM, D_2_O 10%. The reaction was stopped by addition of EDTA 11 mM and heating for 5 min at 60 °C. The samples were then quickly cooled and stored at −20 °C until NMR analysis.

^31^P-NMR spectra were acquired as described in the appropriate paragraph and the content of the substrate and/or the product of the enzyme reaction was measured by integrating the area of the signal. CP 0.5 mM was used as an internal standard. Quantitative results are the average of two independent experiments.

Phosphomannomutase activity was assayed by incubating 0.12 μg PMM2 at 32 °C with 1 mM αM1P as substrate and αG16P or βG16P (5 or 50 μM) or a combination of both, as the activator. Samples were collected over a period of 30 min.

Phosphatase activity was assayed by incubating 42 μg PMM1 at 32 °C for 90 min with αG16P or βG16P 145 μM or a combination of both; at t0 (0′) and at t1 (90′) samples were collected. Each condition was also tested in the presence of IMP 170 μM.

### 4.9. Thermal Shift Assay

Thermal shift assay was carried out as described [50]. In particular wt-PMM2, F119L-PMM2 or V129M-PMM2 to a final concentration of 0.3 mg/mL were equilibrated in Hepes 20 mM pH 7.5, MgCl2 6.25 mM, NaCl 150 mM, DTT 1.25 mM, Sypro Orange 3x, then distributed in 0.2 mL PCR-strip (20 μL each). The appropriate ligand solution (5 μL) was added and the strips were sealed and heated from 20 to 90 °C with temperature increments of 1 °C/min using a StepOne Real Time PCR System. Ligands αG1P, βG1P, vanadate, αG16P, and βG16P were used to a final concentration of 0.5 mM. Four replicas were run for each condition.

### 4.10. Miscellaneous

Cell viability assay was performed as described in [51] on Hek-293 and Caco2 cells using βG16P 0.25 and 0.5 mM for 18 h.

Proteins were quantified with the Quick Start Bradford (Bio-Rad Laboratories Srl, Milan, Italy) using BSA as the standard [52]. SDS-PAGE was performed as in the standard procedures [53].

## Figures and Tables

**Table 1:** Phosphatase activity monitored by ^31^P-NMR. A total of 40 μg PMM1 were incubated for 90 min at 32 °C with 145 μM αG16P or βG16P or a combination of both, in the absence (−) or the presence (+) of inosine monophosphate (IMP) 170 μM.

Bisphosphate (145 μM)	IMP (170 μM)	Residual αG16P (%)	Residual βG16P (%)
αG16P	-	54.2 ± 1.6	-
βG16P	-	-	62.5 ± 7.0
αG16P + βG16P	-	53.3 ± 1.3	93.6 ± 1.2
αG16P	+	0	-
βG16P	+	-	65.7 ± 11.4
αG16P + βG16P	+	0	74.6 ± 3.1

## Abbreviations

CP	creatine phosphate
DTT	dithiothreitol
αG1P	α-glucose-1-phosphate
αG16P	α-glucose-1,6-bisphosphate
βG16P	β-glucose-1,6-bisphosphate
IMP	inosine monophosphate
αM1P	α-mannose-1-phosphate
M6P	mannose-6-phosphate
PC	pharmacological chaperone
